# Comparison of Actual Performance in the Flow and Fraction of Inspired O_2_ among Different High-Flow Nasal Cannula Devices: A Bench Study

**DOI:** 10.1155/2021/6638048

**Published:** 2021-05-04

**Authors:** Yuyan Zhou, Zhong Ni, Yuenan Ni, Binmiao Liang, Zongan Liang

**Affiliations:** Department of Respiratory and Critical Care Medicine, West China Hospital, Sichuan University, No. 37 Guoxue Alley, Chengdu 610041, Sichuan, China

## Abstract

**Background:**

High-flow nasal cannula (HFNC) oxygen therapy has been recommended for use in coronavirus disease 2019 (COVID-19) patients with acute respiratory failure and many other clinical conditions. HFNC devices produced by different manufacturers may have varied performance. Whether there is a difference in these devices and the extent of the differences in performance remain unknown.

**Methods:**

Four HFNC devices (AIRVO 2, TNI softFlow 50, HUMID-BH, and OH-70C) and a ventilator with an HFNC module (bellavista 1000) were evaluated. The flow was set at 20, 25, 30, 35, 40, 45, 50, 60, 70, and 80 L/min, and the FiO_2_ was set at 21%, 26%, 30%, 35%, 40%, 45%, 50%, 60%, 70%, 80%, and 90%. Then, one side of the cannulas was clipped to simulate the compression, bending, or blocking of the nasal cannulas. The flow and FiO_2_ of the delivered gas were recorded and compared among settings and devices.

**Results:**

The actual-flow and actual-FiO_2_ delivered by different settings and devices varied. AIRVO 2 had superior performance in flow and FiO_2_ accuracy. bellavista 1000 and OH-70C had good performance in the accuracy of actual-flows and actual-FiO_2,_ respectively. bellavista 1000 and HUMID-BH had a larger flow range from 10 to 80 L/min, but only bellavista 1000 could provide a stable flow with an excessive resistance up to 60 L/min. TNI softFlow 50 had the best flow compensation and could provide sufficient flow with excessive resistance at 20–50 L/min.

**Conclusions:**

The variation in flow, FiO_2_ settings, and devices could influence the actual-flow and actual-FiO_2_ delivered. AIRVO 2 and OH-70C showed better FiO_2_ accuracy. TNI softFlow 50, bellavista 1000, and HUMID-BH could lower the risk of insufficient flow support due to accidental compression or blocking of the cannulas. In addition, ventilators with HFNC modules provided comparable flow and FiO_2_ and could be an alternative to standalone HFNC devices.

## 1. Introduction

High-flow nasal cannula (HFNC) oxygen therapy can deliver heated and humidified high-flow oxygenated gas via nasal cannulas with flow ranges from 10 to 80 L/min in adults depending on the manufacturer. Such flow can cover the physiological inspiratory flow needed by patients to achieve a stable fraction of inspired O_2_ (FiO_2_) of 21–100%. Previous studies have shown that HFNC therapy is easy to apply and prevents nasal epistaxis [1, 2]. In addition, HFNC therapy has multiple physiological advantages, including generating positive end-expiratory pressure (PEEP) [3], washing out dead space [4], decreasing inspiratory effort [3, 5], and improving lung volume and compliance [5]. Evidence suggests that HFNC therapy has some benefits in providing better oxygenation than conventional oxygen therapy (COT) [6, 7]. HFNC therapy could reduce intubation-related adverse events when used as a preoxygenation device [8]. In addition, HFNC therapy decreased the intubation rate but did not impact mortality in patients with acute hypoxemic respiratory failure [9]. Another study showed that HFNC therapy can reduce 90-day mortality compared with noninvasive ventilation (NIV) [10]. Among cardiothoracic surgery patients, compared with NIV, HFNC therapy did not result in a worse rate of reintubation [11]. HFNC therapy also reduced the reintubation rate compared with COT after extubation in lower-risk respiratory failure patients [12]. A recent observational study showed that, in acute hypercapnic exacerbation of chronic obstructive pulmonary disease (AECOPD) patients, HFNC therapy was effective in improving the dyspnea score, gas exchange, and mucus production [13]. HFNC therapy is noninferior to NIV as initial ventilatory support for CO_2_ clearance in mild-to-moderate AECOPD patients [14]. Recently, HFNC therapy has been recommended for use in coronavirus disease 2019 (COVID-19) patients with acute respiratory failure (ARF) [15].

Most of the previous studies in this field focused on exploring how parameter settings affect physiological effects, such as actual inhaled FiO_2_ [16, 17], humidification [18–21], the PEEP effect [17, 22, 23], and dead space flushing [23–27], on cylinder or 3D-printed models and even human volunteers [28]. According to previous studies, flow plays a determinant role in the physiological effects of HFNC therapy. HFNC devices produced by different manufacturers may have varied performance, especially in terms of flow delivery. Few studies have compared different HFNC devices by the actual-flow and actual-FiO_2_ at the cannulas. The degree of the difference among the manufacturers remains unknown, and therefore, it is meaningful to compare different devices. Furthermore, the results might provide clinicians with a deeper understanding of the difference among settings and devices to help select the most suitable equipment for the specific clinical condition.

## 2. Materials and Methods

### 2.1. Equipment and Instruments

Four HFNC devices (AIRVO 2, Fisher & Paykel Healthcare, Auckland, New Zealand; TNI softFlow 50, TNI Medical AG, Würzburg, Germany; HUMID-BH, RESPIRACARE, Shenyang, China; OH-70C, Microme, Hunan, China) and a ventilator with HFNC module (bellavista 1000, Imtmedical, Buchs, Switzerland) were included and tested using their own breathing circuits, humidification chambers, nasal cannulas, and other accessories. Details are shown in Table 1.

The Medical Intensive Care Unit (MICU) of the West China Hospital provided the high-flow equipment.

A typical HFNC system comprises a flow generator, active heated humidifier, single-limb heated circuit, and nasal cannula [29]. The international standard for HFNC equipment, particularly the requirements for basic safety and essential performance of ventilatory high-flow therapy equipment (ISO/DIS 80601-2-90), is still under development by the International Organization for Standardization. The flow-generating mechanism and structure of these devices in this study are summarized in Table 2.

A VT PLUS HF gas flow analyzer (Fluke Biomedical) was used to measure the actual-flow rate of the gas delivered by HFNC devices. A MaxO_2_ oxygen analyzer (Maxtec) was used to measure FiO_2_. Beijing Aerospace Changfeng Co., Ltd. provided the instruments mentioned above.

### 2.2. Preparations

**Figure 1:**
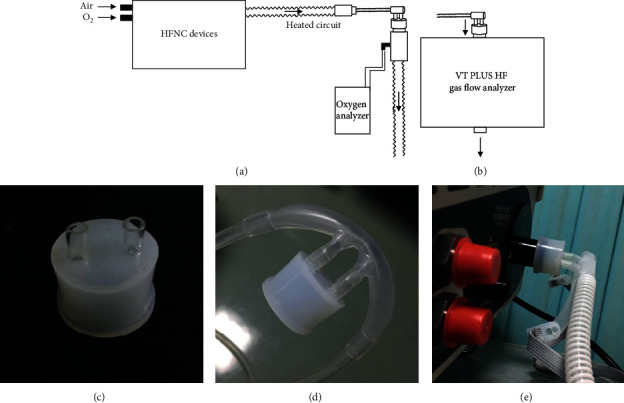
HFNC devices' connection. (a) Connection with an oxygen analyzer. (b) Connection with a flow analyzer. (c) Adapter. (d) Adapter connected to cannulas. (e) Adapter connects cannulas and VT PLUS.

A specially designed adapter in Figure 1 helps to measure the parameters directly from the interface. Two 6 × 4 PU tubes provide ports for nasal cannulas and standardize the different diameters of different interfaces. The 22 mm cap allows attachment to different measuring instruments. This structure does not change the direction of the flow. Thus, the measuring instruments can record the most real performance of these HFNC devices.

For FiO_2_ measurement, cannulas were attached to a conventional ventilation limb, and a sampling tube allowed a small flow of delivered gas to pass through the oxygen analyzer. As Figure 1 shows, tests were performed separately because sampling for FiO_2_ measurement can produce a side flow that may affect the flow measurement.

Tests were performed in a ward of the Medical Intensive Care Unit, West China Hospital. The environmental temperature was controlled during the test procedure. Any equipment that might influence the testing was removed from the room. All HFNC devices and measuring instruments were adequately preheated and calibrated before testing.

### 2.3. Protocol

All the testing processes were repeated three times for FiO_2_ testing and two times for flow testing at different times to reduce disturbance from possible environmental changes and avoid contingency.

HFNC devices were set at 31°C, and the MR850 heated humidifier was set in noninvasive mode. As excess water vapor would affect the accuracy of the VT PLUS gas flow analyzer and MaxO_2_ oxygen analyzer, no water was added to the humidification chamber during FiO_2_ and flow measurements.

The flow was set at 20, 25, 30, 35, 40, 45, 50, 60, 70, and 80 L/min depending on their maximum flow, which was named set-flow. At each level of set-flow, the FiO_2_ was set at 21%, 26%, 30%, 35%, 40%, 45%, 50%, 60%, 70%, 80%, and 90%, which was named set-FiO_2_.

The FiO_2_ and flow rate of the delivered gas from the cannulas were named actual-FiO_2_ and actual-flow, respectively. For each setting combination, after stabilization for 1 min, the actual-FiO_2_ and actual-flow were recorded 3 times at an interval of 10 seconds. Then, one side of the cannulas was clipped to increase the resistance of the nasal cannulas. After another 1 min of stabilization, the clipped-flow was recorded in the same way to estimate the ability to provide sufficient flow under extreme situations.

### 2.4. Statistical Analysis

Normally distributed variables are expressed as the mean ± SD, and nonnormally distributed variables are expressed as the median (interquartile range). The Kruskal–Wallis H test was used to compare the effect of different set‐FiO_2_ on actual-flows and set‐flows on actual-FiO_2_ in a single device. We also used the Kruskal–Wallis H test to compare the actual parameters in different devices under the same settings. The Wilcoxon signed rank test was used to compare differences between settings and actual parameters. The analysis mentioned above can help us to learn the output accuracy of each tested device. The Wilcoxon rank test was used to compare actual-flows with clipped-flows at the same settings to show the effect of increased resistance (clipping one side of the cannulas) on actual-flows in each device.

All statistical tests were 2-sided, and *P* < 0.05 was considered statistically significant. All statistical analyses were performed using IBM SPSS statistical software version 23 for Windows.

## 3. Results and Discussion

### 3.1. Flow

#### 3.1.1. Difference between Actual-Flows and Set-Flows in a Single Device and Different Devices

**Figure 2:**
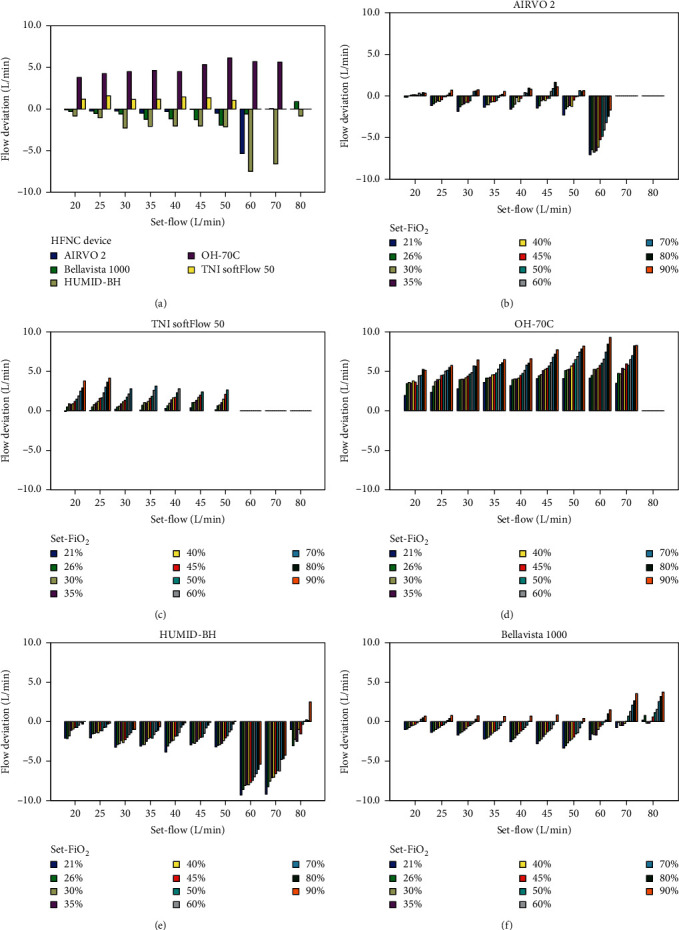
The flow deviation at different set-flow levels in five devices (a). Flow deviation at different set-FiO_2_ levels when set at different set-flow levels in AIRVO 2 (b), TNI softFlow 50 (c), OH-70C (d), HUMID-BH (e), and bellavista 1000 (f). The flow deviation equals the actual-flow minus the corresponding set-flow.

As Figure 2(a) shows, there were significant differences between the actual-flows and the corresponding set-flows in all five devices at most set-flow levels (*P* < 0.001). AIRVO 2 had the minimum difference between actual-flows and set-flows among the five devices (Figure 2) (Table 3).

#### 3.1.2. Influence of Set-FiO_2_ on Actual-Flows in a Single Device

The influence of different settings on actual-flows in different devices is demonstrated in Supplemental Table 1 and Figure 2. An increase in set-FiO_2_ caused a significant increase in actual‐flow at all set-flow levels in TNI softFlow 50, OH-70C, HUMID-BH, and bellavista 1000 (*P* < 0.001) but not AIRVO 2. For AIRVO 2, when set‐flows were fixed at 20 and 25 L/min (*P*=0.99 and 0.5), there was no statistically significant change in actual-flow, while set-FiO_2_ increased (Figure 2(b); Table 3). Another interesting finding was that changes in set-FiO_2_ influenced the actual-flow more significantly at higher set-flows in AIRVO 2 and TNI softFlow 50 (Figures 2(b) and 2(c)).

#### 3.1.3. Difference between Actual-Flows and Clipped-Flows in a Single Device and Different Devices

There were significant differences in actual-flow before and after one side of the cannulas was clipped at any set-flow levels in all devices (*P* < 0.001) but not TNI softFlow 50. The flow change rate was defined as the change in flow after one side of the cannula was clipped and calculated as the actual-flow minus the clipped-flow and then divided by the actual-flow. Notably, TNI softFlow 50 had the best ability to provide the desired flow (flow change rate 0 ± 0.28%) and could almost fully compensate for the flow at various levels of set-flow (20–50 L/min) (Table 4).

**Figure 3:**
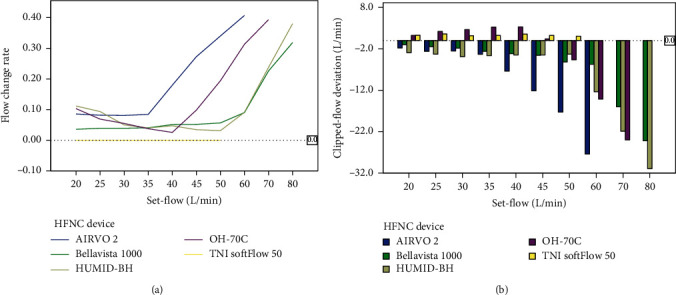
Changes in flow after clipping in different devices. (a) Flow change rate in five devices. (b) Clipped-flow deviation in five devices. The flow change rate was calculated as the actual-flow minus the clipped-flow and then divided by the actual-flow. Clipped-flow deviation equals clipped-flow minus the corresponding set-flow.

When the resistance increased by clipping one side of the cannulas, all five devices could provide a stable flow (flow change rate smaller than 10%) at low set-flow levels but presented different compensatory abilities at higher set-flow levels (Figure 3). When the set-flow was higher than 40 L/min, the flow change rate increased significantly.

### 3.2. FiO_2_

#### 3.2.1. Difference between Actual-FiO_2_ and Set-FiO_2_ in a Single Device and Different Devices

**Figure 4:**
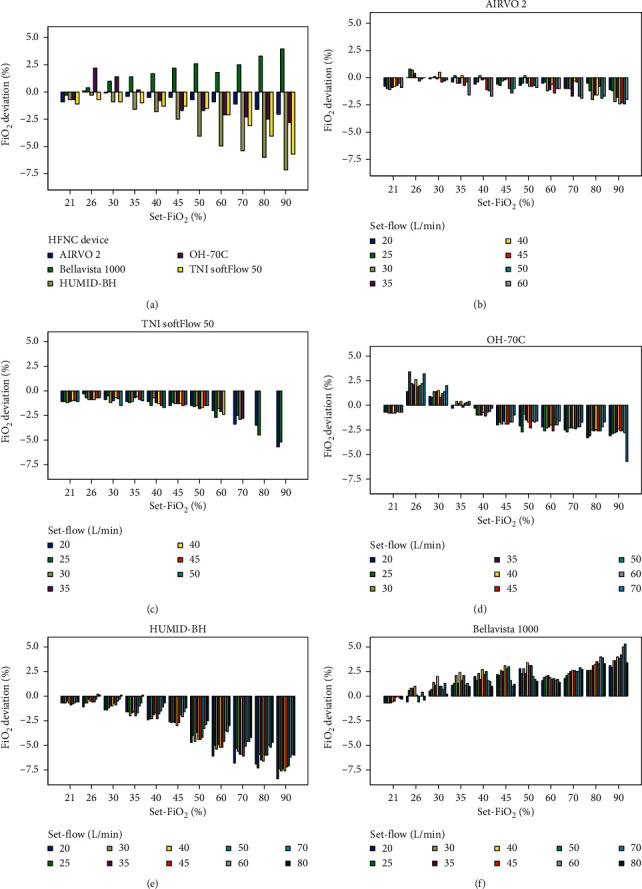
The FiO_2_ deviation at different set-FiO_2_ levels in five devices (a). The FiO_2_ deviation at different set-flow levels when set at different set-FiO_2_ levels in AIRVO 2 (b), TNI softFlow 50 (c), OH-70C (d), HUMID-BH (e), and bellavista 1000 (f). The FiO_2_ deviation equals the actual-FiO_2_ minus the corresponding set-FiO_2_.

The influence of different settings on actual-FiO_2_ in different devices is demonstrated in Supplemental Table 2. There was a significant difference between the actual-FiO_2_ and set-FiO_2_ in all five devices at all set-flow levels (*P* < 0.05 for all), except for AIRVO 2 at 30% (*P*=0.717) (Figure 4). In addition, the actual-FiO_2_ values of different devices presented significant differences at all set-FiO_2_ levels (*P* < 0.001).  Figure 4(a) and Table 5 show that the actual-FiO_2_ of AIRVO 2 were the closest to the set-FiO_2_, while the actual-FiO_2_ of the other four devices were lower or higher.

#### 3.2.2. Influence of Set-Flow on Actual-FiO_2_ in a Single Device

Actual-FiO_2_ of AIRVO 2, TNI softFlow 50, and HUMID-BH showed a trend that, with the increase in set-FiO_2_ and set-flow, the difference between the actual-FiO_2_ and set-FiO_2_ became greater (Figures 4(b), 4(c), and 4(e)). Another interesting point was that the actual-FiO_2_ of these devices were less affected by set-flows at several specific set-FiO_2_ levels (Table 5).

### 3.3. Discussion

This study provides new insights into the changes and quantitative analysis of the actual output of each device under different settings. Among the five devices evaluated, the variation in flow settings, FiO_2_ settings, and devices can influence the actual-flow and actual-FiO_2_ delivered. The following points were obtained:

1. There were significant differences between the actual and set values of flow and FiO_2_. Chikata et al. tested Optiflow (Fisher & Paykel Healthcare) and found a similar trend: as the set-FiO_2_ increased, the differences between the actual-FiO_2_ and the set-FiO_2_ increased [16]. When set-FiO_2_ was fixed, changes in set-flow would also affect the actual-FiO_2_. The air-oxygen mixer might be the reason behind the influence of different FiO_2_ values on the actual-flow and vice versa and the deviation of the measured flow and FiO_2_ with the set-flow and FiO_2_. Changes in settings would alter the input flow or the air and oxygen pressure, which will challenge the air-oxygen mixer and cause an inaccurate output. The variation in oxygen flow below a certain threshold (decided by the accuracy of the built-in flow sensor and algorithm) might not trigger the adjustment of the air flow, resulting in the deviation of the final flow output.
2. TNI softFlow 50, bellavista 1000, and HUMID-BH showed a better ability to provide the desired flow when the resistance abnormally increased. Although the adjustment process was a complex systematic work, the performance of the turbine was the main determining factor.
3. AIRVO 2 had the best performance in the accuracy of actual-flow and actual-FiO_2_. In addition to the air-oxygen mixer factor discussed above, the difference in the FiO_2_ titration target can also partly explain the different performances of FiO_2_ accuracy in manual adjustment devices (AIRVO 2, TNI softFlow 50, and HUMID-BH). In AIRVO 2, the real-time FiO_2_ and flow were measured by an ultrasonic oxygen analyzer, which formed a feedback regulation and achieved the best accuracy. If the real-time FiO_2_ is calculated by the flow of oxygen and air, the concentration of oxygen supply, which is considered to be 100% but usually is not, may cause a deviation in FiO_2_ output. The sensitivity and vulnerability of the oxygen analyzers might also account for the difference between the actual-FiO_2_ and the set-FiO_2_. Dai et al. [30] found that there was no significant difference in actual-FiO_2_ and actual-flow between AIRVO 2 and HUMID-BH under various test conditions. However, in Dai's study, FiO_2_ in both devices was titrated according to the oxygen analyzer (OxiQuant B, Envitec Corporation) at the end of the heated circuit.
4. Bellavista 1000, a ventilator with a built-in high-flow module, performed well in flow accuracy and was secondary to AIRVO 2. From the structure point, ventilators have the same or even a more advanced high-flow generator and air/oxygen mixing equipment as standalone HFNC devices. Ventilators with HFNC modules could provide comparable flow and FiO_2_ and be an alternative to standalone HFNC devices.

Bench evaluation in clinical conditions provides an informative way to assess the actual performance of HFNC devices. The difference in measured flow and FiO_2_ with set values might mislead clinicians to overestimate or underestimate the patient's condition. The clinical consequences of these differences cannot be ruled out and need to be considered. More bench and clinical studies are needed to determine and quantify the degree and consequences of these differences. Increasing the number of devices tested, improving the airway models in bench studies, or conducting clinical tests in healthy volunteers or representative patients are important. However, though clinical studies can make the testing of a wide range of flow and FiO_2_ levels on the same patient more applicable in the clinical setting, it would be ethically questionable to do so.

Finally, FiO_2_ monitoring and feedback regulation are recommended for future HFNC devices. This technique ensures the output accuracy and improves the reliability and safety of the treatment based on our results in AIRVO 2.

There are several limitations in this study. First, there were unavoidable individual differences in the devices we evaluated. There were inevitable possible sample differences in the same model and manufacturer as well. Second, we did not use water for all devices, which was not representative of the clinical use of HFNC devices. The gas was heated but not humidified, and how the absence of humidification and water vapor affected the testing results remains unknown. Future studies are needed to investigate this influence. All the tests were performed at 31°C, and no water was added to achieve accurate flow measurements and minimal equipment aging. According to the ideal gas equation, the measured flow may be higher in actual scenes at high setting temperatures. Third, the environmental temperature and humidity in the ward might affect the results.

## 4. Conclusions

The variation in flow, FiO_2_ settings, and devices can influence the actual-flow and actual-FiO_2_ delivered. The clinical consequences of the deviation cannot be ruled out and need to be considered. AIRVO 2 and OH-70C showed better FiO_2_ accuracy. TNI softFlow 50, bellavista 1000, and HUMID-BH could lower the risk of insufficient flow support due to accidental compression, bending, or blocking of the nasal cannulas. Ventilators with HFNC modules could provide comparable flow and FiO_2_ and be an alternative to standalone HFNC devices.

## Figures and Tables

**Table 1:** Details of HFNC devices.

	TNI softFlow 50	AIRVO 2	HUMID-BH	OH-70C	Bellavista 1000
Heated humidifier	Built-in	Built-in	Built-in	Built-in	Fisher & Paykel MR850
Temperature setting	30, 31, 32, 33, 34, 35, 36, 37°C	31, 34, 37°C	31, 34, 37°C	31, 32, 33, 34, 35, 36, 37°C	Noninvasive mode (31°C), invasive mode (37°C)
Humidification chamber	INTERSURGICAL AF2310	Fisher & Paykel MR290	RESPIRACARE autofill humidification chamber HC-B1	Flexicare autofill humidification chamber 038-31-740CH	INTERSURGICAL AF2310
Breathing circuit	TNI softFlow applicator clinic (large)	900PT501 tube	AIRT-B1-III	Micomme H-180	INTERSURGICAL HFOT single-limb circuit
Nasal cannulas	TNI softFlow applicator clinic (large)	F&P optiflow OPT844 (medium)	RESPIRACARE NAC-1 M	Flexicare Veoflo High-Flow Nasal Cannula (small)	Nasal cannula HFN-0002 (medium)
Flow setting (range, minimum increment)	10–50 L/min, 0.5 L/min	10–25 L/min, 1 L/min; 25–60 L/min, 5 L/min	10–40 L/min, 1 L/min 40–80 L/min, 5 L/min	10–25 L/min, 1 L/min 25–70 L/min, 5 L/min	2–80 L/min, 1 L/min
FiO_2_ setting	Oxygen flowmeter (21–100%, 1%)	Oxygen flowmeter (21–100%, 1%)	Oxygen flowmeter (21–100%, 1%)	Software setting (21–100%, 1%)	Software setting (21–100%, 1%)

**Table 2:** The structure and mechanism differences among devices.

Devices	Air supply^a^	Oxygen supply^b^	Air-oxygen mixer^c^	FiO_2_ monitor^d^
AIRVO 2	Integrated turbines	Low-pressure oxygen supply from separated oxygen flowmeters	Manually adjusts oxygen flow to titrate the aimed FiO_2_ based on monitored or calculated FiO_2_ in real time	Ultrasonic oxygen analyzer
TNI softFlow 50				Not mentioned in public information
HUMID-BH				
OH-70C		Wall supply	Proportional valves	FiO_2_ monitor provided
Bellavista 1000	Wall supply			

Note: ^a^Air supply may be responsible for flow accuracy. ^b^Oxygen supply would affect the FiO_2_ accuracy. Flowmeters cannot provide enough oxygen flow when higher FiO_2_ at high-flow levels is needed. ^c^Changes in settings would alter the input air and oxygen flow or pressure, which will challenge the air-oxygen mixer and cause an inaccurate output. ^d^The FiO_2_ monitor influences the FiO_2_ accuracy.

**Table 3:** Characteristics of the actual-flow in five devices.

Devices	Flow deviation (L/min)			Effect of set-FiO_2_
	≤40 L/min	45–60 L/min	>60 L/min
AIRVO 2	−0.2 (−0.8, 0.3)	−0.9 (−3.6, 0.1)	N/A	No effect at 20 and 25 L/min; effect increase at higher set-flows
Bellavista 1000	−0.7 ± 0.848	−1.4 (−2.2, −0.3)	0.6 (−0.1, 2.2)	No specific pattern
TNI softFlow 50	1.3 (0.8, 2.3)	1.2 (0.8, 2)	N/A	Effect increase at higher set-flows; more obviously than in AIRVO 2
HUMID-BH	−1.6 (−2.4, −0.9)	−2.7 (−6.7, −1.6)	−3.6 (−6.6, −0.8)	No specific pattern
OH–70C	4.4 (3.9, 5.2)	5.7 (5.1, 7.1)	5.7 (5, 6.8)	No specific pattern

The data are presented as the median (IQR) and mean ± SD. The flow deviation equals the actual-flow minus the corresponding set-flow.

**Table 4:** Characteristics of the flow change rate in five devices.

Devices	Flow change rate (%)		
	≤35 L/min	40–60 L/min	>60 L/min
TNI softFlow 50	0 (−0.3, 0)	0 (0, 0.2)	N/A
Bellavista 1000	3.9 (3.6, 4.1)	5.4 (5, 7.9)	28.2 (22.6, 31.9)
HUMID-BH	6.9 (4.2, 10.2)	4.3 (3.2, 7.5)	30.6 (23.8, 38)
OH-70C	5.8 (4.1, 8.6)	14.4 (6.3, 26.9)	39.3 (37.7, 42.3)
AIRVO 2	8.3 (7.1, 9.9)	31.4 (23.3, 38.3)	N/A

The data are presented as the median (IQR). The flow change rate refers to the change in flow after one side of the cannulas is clipped.

**Table 5:** Characteristics of actual-FiO_2_ in five devices.

Devices	≤40%	FiO_2_ deviation (%) 45%–50%	>50%	Effect of set-flow
AIRVO 2	−0.4 (−0.8, 0.1)	−0.6 (−0.9, −0.1)	−1.4 (−2, −0.8)	Best accuracy at 40 L/min^a^
TNI softFlow 50	−1 (−1.2, −0.7)	−1.5 (−1.7, −1.3)	−2.8 (−4, −2.4)	No effect at 26%, 30%, and 50%^b^; cannot reach higher FiO_2_ at high-flow levels^c^
OH-70C	0.3 (−0.7, 1.5)	−1.7 (−2, −1.4)	−2.4 (−2.7, −2.2)	Best accuracy at 35%^d^; effect increased at 60–70 L/min
Bellavista 1000	0.9 (0, 1.6)	2.5 (1.9, 3)	2.6 (2.1, 3.5)	Minor effect at 26%^e^
HUMID-BH	−0.9 (−1.6, −0.5)	−3 (−4.1, −2.5)	−5.9 (−6.8, −5)	Varied effect with no obvious pattern

The data are presented as the median (IQR). The FiO_2_ deviation equals actual-FiO_2_ minus the corresponding set-FiO_2_. ^a^When the set-flow rate was 40 L/min and set-FiO_2_ was 26%, 35%, 40%, 60%, and 70% (*P* < 0.488, 0.857, 0.322, 0.37, and 0.372, respectively), actual-FiO_2_ was not significantly different from the corresponding set-FiO_2_. ^b^When set-FiO_2_ was fixed at 26%, 30%, and 50%, actual-FiO_2_ under different set-flows had no significant difference in TNI softFlow 50 (*P*=0.700, 0.155, 0.166). ^c^The control system of TNI softFlow 50 stopped the rising process, triggering the “oxygen flow is too high” alarm. The alarm cannot be eliminated until the oxygen flow is lowered. ^d^When set-FiO_2_ was 35%, most of actual-FiO_2_ at varied set-flows were not significantly different compared with set-FiO_2_ (*P*=0.439, 0.833, 0.103, and 0.37 for set-flow = 20, 25, 30, 45, and 60 L/min, respectively). ^e^When set-FiO_2_ was 26%, most of actual-FiO_2_ at varied set-flows were not significantly different compared with set-FiO_2_ (*P*=0.37, 0.37, 0.857, and 0.713 for set-flow = 20, 25, 50, 60, and 70 L/min, respectively).

## Data Availability

The data used to support the findings of this study are included within the supplementary information files.

## Supplementary Materials

Supplementary Materials Supplemental Table 1: effects of set-flow, set-FiO_2_, and devices on actual-flow. Supplemental Table 2: effects of set-flow, set-FiO_2_, and devices on actual-FiO_2_.
