# Supplementary material for: Comparison of Actual Performance in the Flow and Fraction of Inspired O2 among Different High-Flow Nasal Cannula Devices: A Bench Study
Source: Can Respir J. 2021 May 4;2021:6638048. doi: 10.1155/2021/6638048 (PMC8112956; doi:10.1155/2021/6638048)
Supplement: Supplementary Materials — Supplemental Table 1: effects of set-flow, set-FiO2, and devices on actual-flow. Supplemental Table 2: effects of set-flow, set-FiO2, and devices on actual-FiO2. [file 6638048.f1.docx]

## Supplementary Materials

## Supplemental Table 1 Effects of set-flow, set-FiO_2_ and devices on actual-flow.

## Supplemental Table 2 Effects of set-flow, set-FiO_2_ and devices on actual-FiO_2_.

| **Supplemental table 1** Effects of set flow, set FiO2 and systems on actual-flow. | | | | | |  |  |
| --- | --- | --- | --- | --- | --- | --- | --- |
| Set- flow | Set-FiO2 | actual-flow of AIRVO 2 (L/min) | actual-flow of TNI softFlow 50 (L/min) | actual-flow of OH-70C (L/min) | actual-flow of HUMID-BH (L/min) | | actual-flow of bellavista 1000 (L/min) |
| 20 L/min | 21% | 19.9 ± 0.126 | 19.9(19.9-19.9) | 22.0(21.1-22.8) | 17.9(17.9-17.9) | | 19.0(19.0-19.0) |
|  | 26% | 19.9 ± 0.082 | 20.5 ± 0.075 | 23.4 ± 0.354 | 17.9(17.4-18.4) | | 19.1(19.0-19.1) |
|  | 30% | 20.0(19.9-20.1) | 20.9 ± 0.075 | 23.6(23.4-23.8) | 18.2(18.0-18.3) | | 19.3(19.2-19.3) |
|  | 35% | 20.1(19.8-20.3) | 20.8(20.8-20.8) | 23.5(23.4-23.7) | 18.9(18.8-19.0) | | 19.5(19.4-19.5) |
|  | 40% | 20.2 ± 0.462 | 20.9(20.8-20.9) | 23.8(23.6-23.9) | 19.1(18.9-19.2) | | 19.6(19.5-19.7) |
|  | 45% | 20.2(19.6-20.8) | 21.2(21.2-21.3) | 23.7(23.6-23.8) | 19.3(18.9-19.6) | | 19.7(19.6-19.8) |
|  | 50% | 20.1(19.7-20.5) | 21.5(21.5-21.5) | 23.2(22.6-23.9) | 19.3(18.9-19.6) | | 19.8 ± 0.121 |
|  | 60% | 20.4 ± 0.677 | 21.9(21.9-21.9) | 24.5(24.4-24.6) | 19.6(19.4-19.7) | | 20.0(19.9-20.1) |
|  | 70% | 20.2(19.6-20.8) | 22.5(22.5-22.5) | 24.5(24.1-24.9) | 19.9(19.7-20.1) | | 20.3(20.1-20.4) |
|  | 80% | 20.4(19.7-21.2) | 22.9(22.9-23.0) | 25.3(24.9-25.5) | 19.7(19.7-19.7) | | 20.5(20.4-20.6) |
|  | 90% | 20.4(19.7-21.0) | 23.8(23.8-23.8) | 25.2(24.8-25.5) | 20.0(20.0-20.0) | | 20.7(20.6-20.8) |
| 25 L/min | 21% | 23.9(23.1-24.7) | 25.1(25.0-25.1) | 27.4(27.0-27.7) | 23.0(22.6-23.3) | | 23.7(23.6-23.7) |
|  | 26% | 24.1(23.3-24.8) | 25.5 ± 0.075 | 28.2(27.3-29.0) | 23.5(23.1-23.8) | | 23.8(23.7-23.9) |
|  | 30% | 24.3(23.4-25.0) | 25.8(25.8-25.8) | 28.7(28.5-28.9) | 23.5(23.3-23.7) | | 24.0(23.9-24.1) |
|  | 35% | 24.4(23.6-25.2) | 26.0 ± 0.075 | 29.0 ± 0.105 | 23.7(23.3-24.0) | | 24.2(24.0-24.2) |
|  | 40% | 24.4(23.5-25.3) | 26.2(26.2-26.2) | 29.0(28.7-29.1) | 23.6(23.2-24.0) | | 24.3(24.2-24.4) |
|  | 45% | 24.6(24.1-25.1) | 26.6(26.6-26.6) | 29.5(29.4-29.6) | 23.9(23.5-24.2) | | 24.5(24.4-24.6) |
|  | 50% | 24.9(24.2-25.6) | 26.7 ± 0.082 | 29.6(29.1-30.0) | 23.9(23.6-24.1) | | 24.6(24.5-24.7) |
|  | 60% | 24.9(24.2-25.6) | 27.3(27.2-27.3) | 30.1(30.0-30.1) | 24.3(23.9-24.6) | | 24.9(24.8-24.9) |
|  | 70% | 25.1(24.3-25.9) | 28.0(28.0-28.0) | 30.2(30.0-30.3) | 24.3(24.3-24.3) | | 25.2(25.1-25.2) |
|  | 80% | 25.4(24.6-26.1) | 28.7(28.6-28.7) | 30.5(30.3-30.7) | 24.7 ± 0.172 | | 25.4(25.3-25.5) |
|  | 90% | 25.7(24.9-26.5) | 29.1 ± 0.082 | 30.7 ± 0.225 | 24.8(24.7-24.8) | | 25.8(25.6-26.0) |
| 30 L/min | 21% | 28.2(27.2-29.1) | 30.2(30.2-30.2) | 32.8(32.3-33.4) | 26.8(26.6-26.9) | | 28.3(28.3-28.3) |
|  | 26% | 28.7(27.9-29.5) | 30.5(30.5-30.5) | 34.0(33.8-34.2) | 27.1(27.0-27.2) | | 28.6(28.5-28.6) |
|  | 30% | 29.0(28.2-29.6) | 30.6 ± 0.279 | 34.0(33.9-34.1) | 27.2 ± 0.190 | | 28.7(28.7-28.8) |
|  | 35% | 29.1(28.3-29.8) | 30.9(30.6-31.1) | 34.0(33.7-34.4) | 27.6(27.5-27.7) | | 28.9(28.9-28.9) |
|  | 40% | 29.3(28.4-30.0) | 31.2 ± 0.225 | 34.2 ± 0.279 | 27.4(27.1-27.6) | | 29.1(29.1-29.1) |
|  | 45% | 29.2(28.4-30.1) | 31.4(31.2-31.5) | 34.4(34.0-34.8) | 27.7(27.7-27.8) | | 29.4(29.3-29.4) |
|  | 50% | 29.4(28.6-30.2) | 31.8(31.7-31.9) | 34.7(34.5-34.8) | 28.0(28.0-28.0) | | 29.5(29.5-29.5) |
|  | 60% | 30.1(29.6-30.5) | 32.2 ± 0.121 | 34.9(34.8-34.9) | 28.4(28.3-28.4) | | 29.7(29.7-29.7) |
|  | 70% | 30.6(30.2-30.9) | 32.8(32.8-32.8) | 35.7 ± 0.261 | 28.6(28.5-28.7) | | 29.9(29.9-29.9) |
|  | 80% | 30.6(30.3-30.9) | N/A | 35.7(35.3-36.1) | 29.0(28.9-29.0) | | 30.3(30.3-30.3) |
|  | 90% | 30.8(30.4-31.1) | N/A | 36.5(36.0-36.9) | 29.0(28.8-29.2) | | 30.8(30.6-30.9) |
| 35 L/min | 21% | 33.7(33.3-34.1) | 35.1 ± 0.117 | 38.6 ± 0.261 | 31.9(31.8-32.0) | | 32.8(32.8-32.8) |
|  | 26% | 34.0(33.4-34.4) | 35.7 ± 0.075 | 39.2 ± 0.315 | 32.1(32.1-32.1) | | 32.9 ± 0.075 |
|  | 30% | 34.0(33.5-34.4) | 36.0 ± 0.082 | 39.2 ± 0.138 | 32.1(31.8-32.5) | | 33.1(33.0-33.1) |
|  | 35% | 34.3(33.8-34.7) | 36.0(35.9-36.1) | 39.3 ± 0.225 | 32.5(32.5-32.5) | | 33.3(33.3-33.3) |
|  | 40% | 34.3(33.7-34.8) | 36.2 ± 0.172 | 39.5 ± 0.354 | 32.9(32.6-33.1) | | 33.6(33.5-33.6) |
|  | 45% | 34.3(34.0-34.6) | 36.6(36.5-36.7) | 39.6(39.3-39.9) | 33.0(32.9-33.0) | | 33.8(33.7-33.8) |
|  | 50% | 34.5(34.2-34.7) | 36.9 ± 0.589 | 39.8(39.4-40.3) | 32.9(32.9-32.9) | | 33.9(33.9-33.9) |
|  | 60% | 34.8(34.3-35.3) | 37.6(37.6-37.6) | 40.3(40.1-40.5) | 33.4(33.3-33.4) | | 34.1(34.1-34.2) |
|  | 70% | 35.1(34.8-35.4) | 38.2(38.0-38.3) | 40.8 ± 0.082 | 33.8 ± 0.082 | | 34.5(34.5-34.6) |
|  | 80% | 35.2(34.9-35.6) | N/A | 41.1(41.1-41.2) | 33.9(33.8-33.9) | | 34.9(34.9-34.9) |
|  | 90% | 35.6(35.0-36.1) | N/A | 41.5 ± 0.387 | 34.4(34.2-34.6) | | 35.6 ± 0.225 |
| 40 L/min | 21% | 38.4(38.0-38.8) | 40.3(40.3-40.3) | 43.2(43.2-43.2) | 36.2(35.8-36.4) | | 37.4 ± 0.082 |
|  | 26% | 38.7(38.0-39.3) | 40.7(40.6-40.7) | 44.0(43.9-44.0) | 36.9(36.9-36.9) | | 37.8(37.7-37.8) |
|  | 30% | 39.0(38.7-39.2) | 41.0(40.8-41.1) | 44.1 ± 0.082 | 37.3 ± 0.137 | | 38.0(37.9-38.0) |
|  | 35% | 39.8(39.5-40.1) | 41.4(41.3-41.4) | 44.1 ± 0.126 | 37.6(37.5-37.7) | | 38.3(38.3-38.3) |
|  | 40% | 39.3(38.7-40.0) | 41.7(41.5-41.7) | 44.2 ± 0.138 | 37.7(37.5-37.9) | | 38.5(38.4-38.5) |
|  | 45% | 39.7(39.2-40.1) | 41.7(41.7-41.7) | 44.5 ± 0.301 | 38.2(37.9-38.4) | | 38.8 ± 0.075 |
|  | 50% | 40.0 ± 0.405 | 42.3(42.2-42.3) | 44.8(44.7-44.8) | 38.2(38.0-38.4) | | 39.0(39.0-39.1) |
|  | 60% | 40.4(39.7-41.0) | 42.8(42.7-42.8) | 45.2 ± 0.138 | 38.6 ± 0.207 | | 39.3 ± 0.082 |
|  | 70% | 40.4(39.4-41.2) | N/A | 45.8(45.7-45.8) | 39.3 ± 0.297 | | 39.6(39.5-39.6) |
|  | 80% | 41.0(40.5-41.4) | N/A | 46.1(45.9-46.2) | 39.6 ± 0.297 | | 40.1(40.0-40.2) |
|  | 90% | 40.8(40.3-41.4) | N/A | 46.6 ± 0.075 | 39.9(39.7-40.0) | | 40.7(40.7-40.8) |
| 45 L/min | 21% | 43.6(42.8-44.3) | 45.4(45.3-45.4) | 49.1(49.0-49.2) | 42.0 ± 0.462 | | 42.2(42.2-42.2) |
|  | 26% | 43.8(43.1-44.6) | 46.1 ± 0.082 | 49.4 ± 0.105 | 42.3(42.1-42.5) | | 42.6(42.4-42.7) |
|  | 30% | 44.4(44.1-44.7) | 46.1(46.1-46.1) | 49.6(49.5-49.6) | 42.3(42.0-42.6) | | 42.8(42.7-42.9) |
|  | 35% | 44.6(43.9-45.1) | 46.4(46.2-46.5) | 50.1 ± 0.172 | 42.5 ± 0.279 | | 43.1(43.1-43.1) |
|  | 40% | 44.5(43.7-45.1) | 46.7(46.7-46.8) | 50.2 ± 0.268 | 42.8(42.5-43.2) | | 43.4(43.4-43.4) |
|  | 45% | 44.7(44.1-45.2) | 47.0(47.0-47.0) | 50.4 ± 0.387 | 43.0(42.4-43.5) | | 43.7(43.7-43.7) |
|  | 50% | 44.7 ± 0.075 | 47.4(47.4-47.4) | 50.7(50.3-51.1) | 43.1 ± 0.589 | | 43.9(43.9-44.0) |
|  | 60% | 45.6 ± 0.333 | N/A | 51.2 ± 0.480 | 43.5(43.1-43.9) | | 44.1(44.1-44.2) |
|  | 70% | 45.9 ± 0.279 | N/A | 51.8 ± 0.514 | 44.2 ± 0.408 | | 44.6(44.5-44.7) |
|  | 80% | 46.7(45.7-47.6) | N/A | 52.2(52.1-52.3) | 44.5 ± 0.415 | | 45.0 ± 0.075 |
|  | 90% | 46.1(45.8-46.4) | N/A | 52.8 ± 0.082 | 44.9(44.8-44.9) | | 45.9(45.7-46.0) |
| 50 L/min | 21% | 47.7(47.5-47.9) | 50.1 ± 0.082 | 54.1 ± 0.172 | 46.8 ± 0.172 | | 46.6 ± 0.082 |
|  | 26% | 48.5(47.7-49.3) | 50.6 ± 0.082 | 55.1 ± 0.075 | 47.0 ± 0.117 | | 47.0(46.7-47.0) |
|  | 30% | 48.7(48.2-49.2) | 50.8(50.6-50.9) | 55.2 ± 0.261 | 47.1 ± 0.075 | | 47.3(47.1-47.4) |
|  | 35% | 48.9(48.6-49.1) | 51.0 ± 0.082 | 55.3 ± 0.172 | 47.2 ± 0.354 | | 47.6(47.6-47.6) |
|  | 40% | 48.8(48.2-49.2) | 51.5 ± 0.297 | 55.7 ± 0.319 | 47.6 ± 0.089 | | 47.8(47.7-47.8) |
|  | 45% | 49.5(49.5-49.5) | 52.1(52.1-52.1) | 56.0 ± 0.387 | 48.0(47.6-48.3) | | 48.0 ± 0.082 |
|  | 50% | 49.9(49.6-50.5) | 52.7(52.4-52.9) | 56.5(56.4-56.6) | 48.2 ± 0.172 | | 48.4 ± 0.138 |
|  | 60% | 50.0 ± 0.315 | N/A | 56.9 ± 0.172 | 48.7(48.3-49.0) | | 48.6(48.4-48.8) |
|  | 70% | 50.7(50.5-50.8) | N/A | 57.4 ± 0.121 | 48.9 ± 0.163 | | 49.2(49.0-49.4) |
|  | 80% | 50.6(49.5-51.5) | N/A | 57.9 ± 0.333 | 49.7 ± 0.172 | | 49.8(49.7-49.9) |
|  | 90% | 50.7(50.0-51.2) | N/A | 58.2 ± 0.075 | 50.1 ± 0.333 | | 50.4(50.2-50.5) |
| 60 L/min | 21% | 53.0(52.1-53.7) | N/A | 64.2 ± 0.248 | 50.7(50.1-51.3) | | 57.7 ± 0.075 |
|  | 26% | 53.6(52.8-54.3) | N/A | 64.5(64.1-64.9) | 51.4 ± 0.408 | | 58.5(58.3-58.5) |
|  | 30% | 53.3(51.9-54.6) | N/A | 65.2 ± 0.105 | 51.9(51.5-52.4) | | 58.4 ± 0.243 |
|  | 35% | 53.4(52.7-54.1) | N/A | 65.2 ± 0.121 | 52.1 ± 0.609 | | 58.3 ± 0.155 |
|  | 40% | 53.9(52.6-55.1) | N/A | 65.4 ± 0.172 | 52.0 ± 0.534 | | 59.0 ± 0.155 |
|  | 45% | 54.8(53.8-55.7) | N/A | 65.8 ± 0.207 | 52.3 ± 0.643 | | 59.4(59.2-59.5) |
|  | 50% | 55.2(54.5-55.8) | N/A | 66.0 ± 0.207 | 52.6(52.4-52.9) | | 59.6(59.6-59.6) |
|  | 60% | 55.9(54.7-57.1) | N/A | 66.6 ± 0.105 | 53.0 ± 0.319 | | 60.0 ± 0.082 |
|  | 70% | 56.8(55.5-58.1) | N/A | 67.4 ± 0.207 | 53.4 ± 0.339 | | 60.2(60.1-60.3) |
|  | 80% | 57.6(56.4-58.7) | N/A | 68.5 ± 0.333 | 54.0(53.6-54.3) | | 61.0(61.0-61.0) |
|  | 90% | 58.3(57.1-59.4) | N/A | 69.3 ± 0.197 | 54.6(54.2-55.0) | | 61.5(61.5-61.5) |
| 70 L/min | 21% | N/A | N/A | 73.5 ± 0.261 | 60.8 ± 0.279 | | 69.2 ± 0.121 |
|  | 26% | N/A | N/A | 74.8 ± 0.207 | 61.8 ± 0.243 | | 69.9(69.7-70.0) |
|  | 30% | N/A | N/A | 74.7 ± 0.261 | 62.4 ± 0.126 | | 69.5(69.2-69.7) |
|  | 35% | N/A | N/A | 75.4(75.4-75.5) | 62.9 ± 0.190 | | 69.5(69.5-69.5) |
|  | 40% | N/A | N/A | 75.2 ± 0.187 | 63.0 ± 0.105 | | 69.8(69.5-69.9) |
|  | 45% | N/A | N/A | 75.9 ± 0.248 | 63.4 ± 0.160 | | 70.0 ± 0.105 |
|  | 50% | N/A | N/A | 75.8 ± 0.240 | 63.8 ± 0.214 | | 70.7 ± 0.103 |
|  | 60% | N/A | N/A | 76.5 ± 0.155 | 63.8(63.6-64.0) | | 71.3(71.3-71.4) |
|  | 70% | N/A | N/A | 77.0 ± 0.190 | 65.2 ± 0.214 | | 72.1 ± 0.234 |
|  | 80% | N/A | N/A | 78.3 ± 0.379 | 65.3 ± 0.197 | | 72.7(72.4-72.8) |
|  | 90% | N/A | N/A | 78.3(78.3-78.4) | 65.8 ± 0.248 | | 73.5 ± 0.126 |
| 80 L/min | 21% | N/A | N/A | N/A | 79.0(79.0-79.1) | | 80.2 ± 0.075 |
|  | 26% | N/A | N/A | N/A | 77.0 ± 0.138 | | 80.8(80.5-81.0) |
|  | 30% | N/A | N/A | N/A | 77.7 ± 0.121 | | 79.8 ± 0.190 |
|  | 35% | N/A | N/A | N/A | 77.5(77.5-77.5) | | 79.8 ± 0.279 |
|  | 40% | N/A | N/A | N/A | 79.0(78.9-79.2) | | 80.0 ± 0.243 |
|  | 45% | N/A | N/A | N/A | 78.4 ± 0.207 | | 80.6 ± 0.297 |
|  | 50% | N/A | N/A | N/A | 79.7 ± 0.126 | | 81.2(80.9-81.3) |
|  | 60% | N/A | N/A | N/A | 80.1(80.0-80.2) | | 81.6(81.3-81.8) |
|  | 70% | N/A | N/A | N/A | 80.3(80.2-80.4) | | 82.6 ± 0.121 |
|  | 80% | N/A | N/A | N/A | 80.2 ± 0.354 | | 83.2 ± 0.075 |
|  | 90% | N/A | N/A | N/A | 82.5 ± 0.339 | | 83.8 ± 0.163 |
| Normally distributed variables were expressed as the mean ± SD, and nonnormally distributed variables were expressed as the median (interquartile range). | | | | | | | |

| **Supplemental table 2** Effects of set flow, set FiO2 and devices on actual-FiO2. | | | | | | |
| --- | --- | --- | --- | --- | --- | --- |
| Set-FiO2 | Set-flow (L/min) | actual-FiO2 of AIRVO 2 (%) | actual-FiO2 of TNI softFlow 50 (%) | actual-FiO2 of OH-70C (%) | actual-FiO2 of HUMID-BH (%) | actual-FiO2 of bellavista 1000 (%) |
| 21% | 20 | 20.2(19.6-20.3) | 19.9 ± 0.205 | 20.3(20.0-20.4) | 20.3 ± 0.368 | 20.4 ± 0.350 |
|  | 25 | 20.0 ± 0.194 | 19.9(19.7-20.5) | 20.3 ± .237 | 20.3 ± 0.296 | 20.3(20.2-20.7) |
|  | 30 | 19.9(19.9-20.0) | 19.8(19.8-20.6) | 20.2(20.0-20.3) | 20.3(19.9-20.6) | 20.3(20.3-20.4) |
|  | 35 | 20.1 ± 0.213 | 19.9(19.9-20.5) | 20.2 ± 0.078 | 20.5(19.9-20.6) | 20.3(20.1-20.4) |
|  | 40 | 20.1 ± 0.252 | 20.0(19.9-20.6) | 20.2 ± 0.127 | 20.3(19.9-20.3) | 20.4(20.4-20.9) |
|  | 45 | 20.2 ± 0.295 | 20.0 ± 0.173 | 20.2(20.2-20.3) | 20.1 ± 0.120 | 20.6 ± 0.299 |
|  | 50 | 20.3 ± 0.347 | 19.9(19.8-20.3) | 20.3 ± 0.156 | 20.2 ± 0.087 | 20.9(20.8-20.9) |
|  | 60 | 20.1 ± 0.250 | N/A | 20.3(20.2-20.3) | 20.3(20.3-20.6) | 21.0(20.9-21.3) |
|  | 70 | N/A | N/A | 20.3(20.3-20.4) | 20.4(20.3-20.4) | 20.8 ± 0.120 |
|  | 80 | N/A | N/A | N/A | 20.4(20.2-20.4) | 20.7(20.7-20.8) |
| 26% | 20 | 26.0 ± 0.196 | N/A | 27.4 ± 0.339 | 24.9(24.6-26.5) | 25.4(25.1-26.8) |
|  | 25 | 26.8(26.4-26.9) | 25.7(25.3-25.8) | 29.6 ± 2.727 | 25.3 ± 0.477 | 26.6(25.3-26.8) |
|  | 30 | 26.7 ± 0.222 | 25.3(25.3-26.2) | 28.2 ± 0.222 | 25.3(25.2-25.8) | 26.8(25.6-26.9) |
|  | 35 | 26.4(26.3-26.4) | 25.1(25.0-26.3) | 28.0 ± 0.471 | 25.8 ± 0.235 | 26.8(26.4-26.9) |
|  | 40 | 26.0(25.5-26.1) | 25.1(25.1-25.3) | 28.6 ± 0.412 | 25.5 ± 0.255 | 27.0(26.9-27.0) |
|  | 45 | 25.8 ± 0.349 | 25.1(25.0-26.2) | 27.9 ± 0.265 | 25.4 ± 0.233 | 26.1(26.0-26.9) |
|  | 50 | 25.9 ± 0.162 | 25.3(24.8-25.3) | 28(27.95-29.1) | 25.4(25.2-26.0) | 25.4(25.3-27.0) |
|  | 60 | 26.0(26.0-26.3) | 25.3(24.8-25.4) | 28.2(28.2-29.4) | 25.7 ± 0.087 | 26.0(25.9-26.7) |
|  | 70 | N/A | N/A | 29.2(28.9-29.2) | 26.2 ± 0.309 | 26.4(25.4-26.6) |
|  | 80 | N/A | N/A | N/A | 26.1(26.0-27.0) | 25.6 ± 0.304 |
| 30% | 20 | 29.9 ± 0.133 | 29.1(29.0-29.6) | 30.8 ± 0341 | 28.6(28.2-28.8) | 30.5(30.3-31.6) |
|  | 25 | 30.0(29.9-31.0) | 29.5(28.6-29.7) | 30.6 ± 1.222 | 28.6 ± 0.354 | 30.7 ± 0.362 |
|  | 30 | 30.0 ± 0.255 | 28.8(28.7-29.5) | 31.3 ± 0.332 | 28.8(28.8-29.2) | 31.4(30.9-31.6) |
|  | 35 | 29.9 ± 0.283 | 29.0(29.0-29.7) | 31.4(31.4-31.6) | 29.0(28.4-29.1) | 31.1(31.1-31.2) |
|  | 40 | 30.5(30.5-30.6) | 29.2 ± 0.626 | 31.5(31.5-31.6) | 29.1 ± 0.354 | 32.0(31.8-32.1) |
|  | 45 | 29.6(29.4-29.6) | 29.2(29.0-29.6) | 30.9 ± 0.354 | 29.3(27.9-29.5) | 31.0(30.9-31.2) |
|  | 50 | 29.7(29.6-30.9) | 28.5(28.5-29.3) | 31.2(31.2-31.8) | 29.1 ± 0.625 | 31.0(30.9-32.1) |
|  | 60 | 29.8(29.7-30.4) | N/A | 31.5 ± 0.710 | 29.4 ± 0.220 | 30.7(30.7-30.8) |
|  | 70 | N/A | N/A | 32.1 ± 0.436 | 29.7(29.6-29.9) | 31.3(30.3-31.4) |
|  | 80 | N/A | N/A | N/A | 30.1(30.1-30.1) | 30.3 ± 0.614 |
| 35% | 20 | 34.6 ± 0.120 | 33.9(33.3-34.1) | 34.8 ± 0.778 | 33.4(32.4-33.5) | 36.1(35.8-37.2) |
|  | 25 | 35.3 ± 0.166 | 33.8(33.5-33.8) | 34.9 ± 0.788 | 33.4(32.8-33.6) | 36.3(36.2-36.3) |
|  | 30 | 34.6 ± 0.196 | 33.9 ± 0.194 | 35.4(35.0-35.5) | 33.0 ± 0.477 | 37.1(36.8-37.2) |
|  | 35 | 34.5(34.5-35.0) | 34.2 ± 0.350 | 35.3 ± 0.259 | 33.3(33.1-33.4) | 36.3(36.3-36.8) |
|  | 40 | 35.2(34.2-35.3) | 34.4 ± 0.276 | 35.5 ± 0.283 | 33.5 ± 0.423 | 37.4 ± 0.078 |
|  | 45 | 34.4 ± 0.207 | 34.1(33.7-34.2) | 34.8 ± 0.364 | 33.0(32.6-33.1) | 36.6(36.5-36.6) |
|  | 50 | 34.6(34.1-34.8) | 34.0 ± 0.222 | 35.2(35.2-35.7) | 33.4 ± 0.614 | 37.1(37.0-37.6) |
|  | 60 | 33.5 ± 0.582 | N/A | 35.4 ± 0.842 | 34.0(33.8-34.5) | 36.1(35.7-36.3) |
|  | 70 | N/A | N/A | 35.4(35.3-35.8) | 34.3(34.3-35.0) | 36.3(35.3-36.4) |
|  | 80 | N/A | N/A | N/A | 35.1(34.7-35.3) | 36.0(35.1-36.0) |
| 40% | 20 | 39.4(39.0-39.5) | 38.9 ± 0.260 | 39.9 ± 1.374 | 37.4 ± 0.789 | 42.1 ± 0.452 |
|  | 25 | 39.6(39.5-39.6) | 38.5(38.4-39.4) | 39.0(38.8-39.2) | 37.6 ± 0.527 | 41.6(41.5-41.6) |
|  | 30 | 40.3 ± 0.300 | 39.2 ± 0.495 | 39.0(39.0-39.4) | 37.7 ± 0.296 | 42.3(42.3-42.5) |
|  | 35 | 39.8 ± 0.194 | 38.8(38.8-39.3) | 39.0(38.9-39.4) | 38.1(37.9-38.2) | 41.7(41.7-42.3) |
|  | 40 | 39.9(39.2-40.2) | 38.7 ± 0.362 | 39.3 ± 0.280 | 38.3 ± 0.308 | 42.7(42.7-42.8) |
|  | 45 | 38.9(38.6-39.7) | 38.5 ± 0.162 | 38.9 ± 0.507 | 37.7 ± 0.650 | 42.2(41.7-42.3) |
|  | 50 | 38.8(38.6-38.8) | 38.3(38.2-38.9) | 39.3(39.1-40.2) | 38.2(38.1-38.6) | 42.5(42.5-42.6) |
|  | 60 | 38.4 ± 0.270 | N/A | 39.4(39.2-40.7) | 38.5(38.4-38.7) | 41.6(41.0-41.7) |
|  | 70 | N/A | N/A | 39.7(39.7-39.8) | 38.9 ± 0.162 | 41.5(40.7-41.6) |
|  | 80 | N/A | N/A | N/A | 39.3 ± 0.779 | 40.9 ± 0.442 |
| 45% | 20 | 44.3 ± 0.696 | 43.5(42.8-43.5) | 43.0(42.5-44.2) | 42.3 ± 0.541 | 47.2 ± 0.304 |
|  | 25 | 44.3 ± 0.218 | 43.8(43.5-44.7) | 43. ± 0.350 | 42.3(42.1-42.4) | 47.1(46.8-47.2) |
|  | 30 | 44.7 ± 0.192 | 43.7(43.7-43.8) | 43.1(43.0-43.2) | 42.4(42.2-42.5) | 47.6(47.5-47.8) |
|  | 35 | 44.8(44.8-45.1) | 43.7(43.1-43.8) | 43.4(43.0-43.7) | 42.3 ± 0.173 | 47.5 ± 0.218 |
|  | 40 | 44.9(44.4-45.0) | 43.7(43.2-43.9) | 43.1(43.1-43.3) | 42.0(41.0-42.4) | 48.1(48.0-48.1) |
|  | 45 | 44.0(43.9-45.8) | 43.5(43.3-44.5) | 43.0 ± 0.350 | 42.3(41.2-42.5) | 47.8(47.2-47.9) |
|  | 50 | 43.6(43.4-44.6) | 43.6(43.4-43.7) | 43.3(43.2-43.7) | 43.1(42.7-43.1) | 48.0(47.9-48.0) |
|  | 60 | 44.0(44.0-45.1) | N/A | 43.3(43.1-44.3) | 42.9(42.8-43.0) | 46.6 ± 0.087 |
|  | 70 | N/A | N/A | 44.0 ± 0.190 | 43.4 ± 0.378 | 46.0(45.9-46.7) |
|  | 80 | N/A | N/A | N/A | 43.8 ± 0.564 | 46.2 ± 0.550 |
| 50% | 20 | 49.3(48.5-49.6) | 48.5(48.0-48.6) | 47.9 ± 0.580 | 45.3 ± 0.408 | 52.7 ± 0.337 |
|  | 25 | 49.5(49.3-49.6) | 48.4(48.1-49.1) | 47.3(47.0-49.3) | 46.0(45.8-46.0) | 52.3 ± 0.247 |
|  | 30 | 50.3 ± 0.310 | 48.5(48.0-48.6) | 49.1(47.9-49.3) | 45.4 ± 0.247 | 52.8(52.7-53.0) |
|  | 35 | 49.5 ± 0.524 | 48.2(48.1-48.5) | 48.5(48.0-48.6) | 46.3(45.6-46.4) | 52.3(52.3-52.6) |
|  | 40 | 49.4 ± 0.621 | 48.3(48.2-48.9) | 48.2 ± 0.235 | 45.4 ± 0.732 | 53.4(53.2-53.4) |
|  | 45 | 49.3 ± 0.124 | 48.5(48.4-49.5) | 47.7(47.5-47.8) | 45.6(44.8-45.9) | 53.1(52.7-53.2) |
|  | 50 | 49.3(49.3-50.6) | 48.5(48.5-48.9) | 48.4(47.9-48.5) | 45.8(45.8-46.0) | 53.1(53.0-53.2) |
|  | 60 | 49.1 ± 0.194 | N/A | 48.4 ± 0.367 | 46.7 ± 0.207 | 52.0(51.9-52.0) |
|  | 70 | N/A | N/A | 48.4(48.3-50.3) | 47.1(46.3-47.3) | 51.7(51.1-52.0) |
|  | 80 | N/A | N/A | N/A | 47.4 ± 0.486 | 51.5(51.2-52.2) |
| 60% | 20 | 59.5(59.5-60.2) | 58.1 ± 0.480 | 57.8(57.6-58.4) | 53.9(53.6-55.0) | 61.6(61.5-62.2) |
|  | 25 | 59.6(59.1-59.6) | 57.3(57.2-57.7) | 57.2 ± 0.803 | 55.0(53.9-55.3) | 61.9(61.8-62.1) |
|  | 30 | 58.8 ± 0.240 | 58.2(58.2-58.5) | 57.7(57.5-58.1) | 54.6 ± 0.122 | 62.0(61.7-62.1) |
|  | 35 | 58.9(57.5-59.0) | 57.8 ± 0.212 | 57.8 ± 0.183 | 55.2 ± 0.212 | 62.1(61.7-62.1) |
|  | 40 | 59.6 ± 0.949 | 57.6(57.5-58.2) | 57.9 ± 0.407 | 54.8(53.4-55.4) | 61.9(61.9-62.0) |
|  | 45 | 58.6(58.0-60.5) | N/A | 57.4(57.4-57.8) | 54.8(53.5-54.9) | 61.7(61.6-62.1) |
|  | 50 | 59.0(58.3-59.3) | N/A | 58.0(57.6-58.1) | 55.4(55.2-55.4) | 61.8(61.8-61.9) |
|  | 60 | 59.1 ± 1.070 | N/A | 58.0(58.0-58.1) | 56.4 ± 0.343 | 61.6(61.6-62.4) |
|  | 70 | N/A | N/A | 58.4 ± 0.162 | 56.4(54.9-56.7) | 61.7(61.6-62.2) |
|  | 80 | N/A | N/A | N/A | 57.0(56.2-57.2) | 61.3 ± 0.395 |
| 70% | 20 | 69.0(67.9-69.3) | 66.6(66.2-66.6) | 67.5(67.5-68.0) | 63.2(63.1-64.1) | 71.8(71.6-72.4) |
|  | 25 | 69.0 ± 0.205 | 67.5(66.8-67.5) | 67.3(67.3-67.7) | 64.7(62.9-65.3) | 72.1 ± 0.346 |
|  | 30 | 69.0 ± 0.824 | 67.1 ± 0.219 | 67.6 ± 0.242 | 64.4(64.4-64.8) | 72.3(71.9-72.3) |
|  | 35 | 68.3 ± 0.665 | 67.2(66.7-67.3) | 67.7 ± 0.187 | 64.2 ± 0.310 | 72.5(71.9-72.6) |
|  | 40 | 69.6 ± 0.774 | N/A | 67.7 ± 0.087 | 64.1(62.8-64.6) | 72.6(72.6-72.6) |
|  | 45 | 69.6(68.4-69.8) | N/A | 67.6(67.4-67.6) | 63.8 ± 1.073 | 72.6(72.5-72.8) |
|  | 50 | 68.2 ± 0.518 | N/A | 67.8(67.6-67.8) | 64.9(64.1-64.9) | 72.5(72.5-72.5) |
|  | 60 | 68.1(67.7-69.4) | N/A | 67.9 ± 0.626 | 65.4(64.9-65.6) | 72.5(72.3-73.5) |
|  | 70 | N/A | N/A | 68.3(68.0-69.0) | 65.4(64.1-65.8) | 72.9 ± 0.218 |
|  | 80 | N/A | N/A | N/A | 65.8 ± 0.509 | 72.8 ± 0.482 |
| 80% | 20 | 79.5(77.9-79.6) | 76.4 ± 0.299 | 77.0 ± 0.555 | 73.1(72.7-74.3) | 82.6(82.3-83.5) |
|  | 25 | 78.8(78.4-80.6) | 75.5(75.4-75.8) | 76.9(76.8-77.2) | 72.7(72.5-74.3) | 82.6 ± 0.362 |
|  | 30 | 78.0(77.9-78.2) | N/A | 77.5(77.5-77.9) | 74.1 ± 0.164 | 82.6(82.6-83.1) |
|  | 35 | 78.4(78.4-78.5) | N/A | 77.3 ± 0.242 | 73.5(72.9-73.6) | 83.1 ± 0.346 |
|  | 40 | 78.4 ± 0.695 | N/A | 77.5 ± 0.242 | 73.2 ± 1.177 | 83.2(83.1-83.5) |
|  | 45 | 79.2(77.7-79.6) | N/A | 77.4(77.4-77.6) | 74.0(71.7-74.5) | 83.5(83.4-83.7) |
|  | 50 | 78.1(76.5-78.7) | N/A | 77.4 ± 0.148 | 74.0 ± 0.255 | 83.3(83.3-83.7) |
|  | 60 | 78.3(77.6-78.7) | N/A | 77.8(77.6-79.0) | 75.0(73.6-75.3) | 84.0(83.3-84.3) |
|  | 70 | N/A | N/A | 78.3(78.0-78.3) | 74.9 ± 0.638 | 84.1 ± 0.364 |
|  | 80 | N/A | N/A | N/A | 75.3(74.0-75.4) | 83.3 ± 0.708 |
| 90% | 20 | 88.9(87.9-89.1) | 84.3(84.0-84.4) | 86.9 ± 0.252 | 81.6(81.0-83.4) | 93.1(92.8-94.0) |
|  | 25 | 88.8 ± 0.205 | 84.6 ± 0.406 | 87.1(87.0-87.9) | 82.5 ± 0.912 | 92.9 ± 0.563 |
|  | 30 | 87.8(87.5-87.9) | N/A | 87.3 ± 0.245 | 82.4(82.4-82.5) | 93.6(93.5-93.7) |
|  | 35 | 88.1 ± 0.442 | N/A | 87.4 ± 0.176 | 82.6(82.0-82.8) | 93.6(93.4-94.0) |
|  | 40 | 87.6(87.4-88.4) | N/A | 87.5 ± 0.260 | 82.2 ± 0.964 | 94.0(94.0-94.1) |
|  | 45 | 87.7(87.4-90.0) | N/A | 87.4(87.1-87.4) | 82.8(81.2-83.0) | 93.8(93.8-93.9) |
|  | 50 | 87.6(87.5-88.5) | N/A | 87.4(87.4-88.6) | 82.9(82.8-83.5) | 94.2(94.2-94.3) |
|  | 60 | 88.0(87.8-88.6) | N/A | 87.2 ± 0.464 | 83.8 ± 0.568 | 95.0(94.9-95.0) |
|  | 70 | N/A | N/A | 84.5 ± 0.430 | 84.0 ± 0.536 | 95.3(94.7-95.4) |
|  | 80 | N/A | N/A | N/A | 84.1 ± 0.568 | 93.4(92.9-95.6) |
| Normally distributed variables were expressed as the mean ± SD, and nonnormally distributed variables were expressed as the median (interquartile range). | | | | | | |
